# A pilot study of nebulized 3D-cultured mesenchymal stem cell-derived extracellular vesicles for the treatment of post-COVID-19 chronic cough

**DOI:** 10.3389/fmed.2026.1809451

**Published:** 2026-07-21

**Authors:** Jihui Du, Yunong Wang, Hongyan Wang, Hongjie Zou, Chu Hua, Weihan Xie, Jiming Chen, Zhijiang Yu, Xilin Shen, Keke Wu, Cheng Zhang, Wenhao Wang, Bingbing Wu, Zhiyong Zhang

**Affiliations:** 1 Research Center for Clinical and Translational Medicine, Shenzhen Nanshan People's Hospital and Affiliated Nanshan Hospital of Shenzhen University, Shenzhen, Guangdong, China; 2 Translational Research Center of Regenerative Medicine and 3D Printing of Guangzhou Medical University, Guangdong, China; 3 Department of Infectious Diseases and Shenzhen Key Laboratory for Endogenous Infection, Shenzhen Nanshan People's Hospital, Shenzhen, China; 4 REGEN-αGEEK Medical Technology Co., Ltd, Shenzhen, China; 5 Department of Respiratory and Critical Care Medicine, Shenzhen Nanshan People's Hospital, Shenzhen, China; 6 Department of Obstetrics and Gynecology, Center for Reproductive Medicine, The Fourth Affiliated Hospital of School of Medicine, and International School of Medicine, International Institutes of Medicine, Zhejiang University, Yiwu, China; 7 School of Biomedical Engineering, Guangzhou Medical University, Guangzhou, China; 8 The Guangdong-Hong Kong-Macau Joint Laboratory for Cell Fate Regulation and Diseases, GMU-GIBH Joint School of Life Sciences, Guangzhou Medical University, Guangzhou, China; 9 The State Key Lab of Respiratory Disease, The First Affiliated Hospital, Guangzhou Institute of Respiratory Disease, Guangzhou Medical University, Guangzhou, China

**Keywords:** 3D culture, extracellular vesicles, immunomodulation, mesenchymal stem cells, nebulization therapy, post-COVID-19 chronic cough

## Abstract

**Introduction:**

Chronic cough, particularly prevalent in post-COVID condition (PCC, also known as long COVID), remains a significant medical challenge. Recently, extracellular vesicles (EVs) have gained significant attention for their therapeutic potential.

**Methods:**

In this study, we explore the therapeutic effects of EVs derived from mesenchymal stem cells (MSCs) cultured in a 3D system (3D-EVs) in treating chronic cough, with a focus on post-COVID-19 patients.

**Results:**

Our *in vitro* experiments demonstrated that 3D-EVs promote angiogenesis and cell migration, crucial processes in tissue repair and regeneration. Notably, 3D-EVs exhibited a robust suppressive effect on lymphocyte proliferation in human PBMCs, indicating their potential immunomodulatory role. At day 6, nebulized 3D-EVs treatment group showed significantly higher rates of significant improvement (22.5% vs. 5.0%, *P* = 0.023) and total effectiveness (67.5% vs. 47.5%, *P* = 0.035), as well as a shorter mean time to cough resolution (13.83 vs. 19.90 days, *P* = 0.037). By day 14, total effective rates were comparable (85% vs. 80%, *P* > 0.05). No safety concerns were observed. RNA sequencing revealed altered B cell receptor signaling and downregulation of the mitotic cell cycle pathway, while exploratory immunophenotyping identified a significant reduction in plasma cells (*P* < 0.05) and directional trends in other immune subsets, providing preliminary evidence that 3D-EVs may modulate B cell differentiation and immune responses.

**Conclusion:**

These findings demonstrate the feasibility and preliminary efficacy of 3D-EVs for rapid relief of post-COVID-19 chronic cough, supporting larger controlled trials to validate their role in this and related respiratory diseases.

## 1 Introduction

MSCs have been used for diverse clinical applications in the treatment of autoimmune diseases due to their immunomodulation potential (1). EVs are small vesicles that can be secreted by various cell types. Owing to their small size with an average diameter of approximately 30~150 nm, they can effectively bypass physiological barriers and reach the intended target tissues and serve as mediators of cell-to-cell communication (2, 3). Moreover, recent studies suggest that EVs secreted from MSCs are critical for mediating the biological functions of MSCs (4). These EVs have emerged as important markers in liquid biopsies, offering significant potential for disease diagnosis, therapeutic interventions, and prognostic assessments (5, 6). The use of MSC-EVs is increasingly being considered as a cell-free therapy option. MSC-EVs offer potential advantages over MSCs, including lower immunogenicity, easier storage and transport, making them attractive for clinical applications (7). MSCs can modulate both adaptive and innate immunity, possessing the dual capacity to suppress immune cell activation and proliferation, while also promoting immune responses through pro-inflammatory factors and chemokines secretion (8–12). MSCs could improve the allergic airway disease by inhibiting the proliferation and function of dendritic cells (DC) which have an immunomodulatory effect, and which are differentiated into T cells and B cells (13–15). The intravenous injection of MSCs significantly reduced eosinophil infiltration in the nasal mucosa and lung tissue of allergic mouse models and improved the degree of airway hypersensitivity and allergic symptoms (16–18).

The COVID-19 pandemic, caused by the novel coronavirus SARS-CoV-2, has had a significant global impact on health. According to the World Health Organization (WHO), approximately 10% to 20% of COVID-19 patients experience persistent symptoms after acute COVID-19 infection (19). Post-COVID condition (PCC) encompasses multiple adverse outcomes, including cardiovascular, type 2 diabetes (20), cerebrovascular disease (21) and dozens of other symptoms across multiple organ systems (22) that may due to immune dysregulation (23). Similar to typical respiratory infections such as the common cold or influenza, coughing stands out as a significant symptom of COVID-19 and has the potential to linger during the post-infection period, reflecting the virus's impact on the respiratory system. Research data suggests that up to 18% of patients develop a persistent cough after COVID-19 infection (24). As the COVID-19 virus primarily invades the respiratory tract mucosa, causing local inflammatory response and airway hyperreactivity, the cough following COVID-19 infection tends to be more severe and persistent. Identifying safe and rapidly acting therapies for post-COVID chronic cough is of clinical importance. Furthermore, patients with chronic cough often seek antitussive therapies, but it is unclear whether these approaches are effective in managing cough in individuals recovering from COVID-19 (25).

MSC derived-EVs (MSC-EVs) have received much attention as a novel cell-free therapeutic strategy during this period. Studies have shown that MSC-EVs can exert therapeutic effects through various pathways including regulating immune response, inhibiting inflammatory response, and alleviating lung injury and inflammation (26–29). Additionally, MSC-EVs can also release multiple biologically active substances, such as cytokines and growth factors (4, 30). Multiple clinical trials on the treatment of lung diseases with MSC-EVs have been registered in the ClinicalTrials.gov database. For example, a randomized, double-blind, placebo-controlled clinical trial named “A Pilot Clinical Study on Inhalation of Mesenchymal Stem Cells Exosomes Treating Severe Novel Coronavirus Pneumonia” (NCT04276987) has been registered on ClinicalTrials.gov. The trial shows a consecutive 5 days inhalation dose of clinical grade haMSC-Exos was feasible and well tolerated in seven COVID-19 patients, with no evidence of prespecified adverse events, immediate clinical instability, or dose-relevant toxicity at any of the doses tested (31). Another clinical trial on nebulization therapy with MSC-derived EVs was conducted on seven patients with COVID-19 pneumonia (ChiCTR2000030261). Nebulization of MSC-derived EVs was found to be a safe and effective method, promoting absorption of pulmonary lesions and reducing hospitalization duration for mild cases of COVID-19 pneumonia (32). Here, we propose a pilot study of nebulized 3D-cultured MSC-EVs for the treatment of chronic cough following COVID-19 infection. This exploratory trial aims to evaluate the safety and preliminary efficacy of this therapy, with a focus on rapid symptom relief and immunomodulatory trends. At the same time, this study is expected to provide initial evidence supporting the feasibility of MSC-EVs for post-COVID chronic cough and to inform the design of larger confirmatory trials.

## 2 Methods

### 2.1 Isolation of mesenchymal stem cells (MSCs)

Clinical-grade MSCs were procured from REGEN-αGEEK MEDICAL TECHNOLOGY CO., LTD (Shenzhen, China, http://www.regengeek.com). Initially, Wharton's jelly was harvested from fresh umbilical cords, which had been stripped of both blood vessels and epidermis. The Wharton's jelly was then sectioned into small pieces to harvest primary cells. Briefly, for the production of 2D-MSCs, primary cells were cultured in UltraMedia^®^ pro serum-free medium (REGEN-αGEEK, RGM0051), supplemented with 1% penicillin/streptomycin (P/S). For the generation of 3D-MSCs, the harvested primary cells, along with biomimetic microcarrier, were cultured in a 500 ml mini-bioreactor for 4 days. This resulted in an approximate 10-fold increase in cell quantity. Subsequently, the MSCs and supernatant were transferred to a 2L bioreactor and cultured for 5 days, yielding another 10-fold increase in cell quantity. The MSCs and supernatant were then collected and transferred to a 10L bioreactor for an additional 6 days of culture. Through this three-stage expansion process, also called 3D culture system, a substantial reserve of supernatant was obtained for EVs extraction.

### 2.2 Isolation of MSC-EVs

2D-EVs and 3D-EVs were obtained from REGEN-αGEEK MEDICAL TECHNOLOGY CO., LTD (Shenzhen, China, http://www.regengeek.com). Briefly, the cell supernatant was centrifuged at 300 g for 10 min to collect the supernatant to remove residual cells. Then, the supernatant was collected by centrifugation of 2,000 g for 10 min to remove cell fragments. And cell fragments and large vesicles were separated by centrifugation at 10,000 g at 4°C for 30 min. After that, the supernatant was transferred to the ultracentrifuge tube and ultracentrifuged (Beckman, America) at 100,000 g for 70 min at 4°C (33). Finally, the MSC-EVs pellet was resuspended in PBS and washed by a second ultracentrifugation at 100,000 g at 4°C for 70 min to eliminate the interference of free protein and impurities. The Critical Quality Control Points of 3D-EVs for clinical use were listed in Table 1.

**Table 1:** Critical quality control points of 3D-EVs.

Parameter	Release criteria	Method
Appearance	Transparent or pale yellow	
pH	6.5~7.5	
Particle analysis	>0.9x10^9^ particles/mL	NTA
Particle size distribution ratio	30~200 nm particles > 80%	NTA
Sterility	Negative	
Endotoxin	< 0.5 EU/mL	
Mycoplasma	Negative	

### 2.3 Characterization of MSC-EVs

Transmission electron microscopy (TEM) was performed as described previously (34). EVs were blotted on glow-discharged (30 s in an Emitech K100X glow discharge system, Quorum Technologies Ltd., Lewes, UK) carbon-coated grids. Grids were washed with PBS for 2 min, fixed with 1% v/v glutaraldehyde for 5 min, and rinsed eight times with double-distilled water for 2 min each. The sample was stained with uranyl oxalate at pH 7 for 5 min and then incubated in a mixture of 2% v/v methylcellulose and 4% v/v uranyl acetate for 10 min on ice. Excess liquid was drained off and the grids were air-dried. The fixed sample was analyzed in a FEI Morgagni 268 microscope (Field Electron and Ion Company, Hillsboro, OR, USA) operated at a 100 kV acceleration voltage in the bright field mode. Size distributions from TEM images were obtained with FIJI software.

For nanoparticle tracking analysis (NTA), EVs distribution and concentration were analyzed using ZetaView Particle Metrix (ZetaView PMX 110). The samples were diluted at 1:1,000 in PBS and analyzed.

For Western blot, MSC-EVs lysates were prepared with RIPA buffer (Gbcbio, G3424), and the protein concentration was measured by BCA assay (Thermo Fisher Scientific). Immunoblot were conducted using the following antibodies: CD9 (Affinity, AF5139), CD63 (Affinity, AF5117), CD81 (Affinity, DF8045), Calnexin (Affinity, AF5362), TSG101 (Proteintech, #67381), GM130 (Thermo Fisher Scientific, PA1-077). Secondary antibodies: Anti-rabbit IgG, HRP-linked antibody (Beyotime, A0208). The signal was developed by ECL (Bio-Rad, 1705060).

### 2.4 Cell scratch assay

Mouse embryo fibroblasts, NIH 3T3 (ATCC CRL-1658) were seeded at a density of 3.5 × 10^4^ cells/well in an iBidi cell migration dish (Procell) and cultured at 37°C under 5% CO_2_. After 24 h, the inserts of the cell migration dishes were removed, and the cells were washed with PBS. Subsequently, the cells were maintained in DMEM supplemented with EVs at concentrations of 0, 20, 40, and 80 μg/mL, along with EVs-depleted FBS (2%). The EVs-depleted FBS was obtained by filtering it using a 100 nm filter and then subjecting it to ultracentrifugation for 16 h. After ultracentrifugation, the FBS was filtered again using a 100 nm filter (35). Images of the scratched areas were captured at 0, 6, 12, 18, and 24 h, and the healing percentage of the wound was calculated using ImageJ software. Each experiment was repeated independently three times.

### 2.5 Tube formation assay

Matrigel (Corning) was used to coat 48-well plates and incubated at 37 °C for 60 min to allow the Matrigel to polymerize. After adding 0, 20, 40 or 80 μg/mL EVs, a total of 4 × 10^4^ HUVECs, kindly provided by REGEN-αGEEK were seeded in the Matrigel-coated wells with 2% EVs-depleted FBS. The plates were then incubated at 37 °C in a 5% CO_2_ humidified atmosphere. After 8 h, tube formation was visualized with a microscope and determined by measuring total numbers of nodes, junctions and the total length of tubes (36). Each experiment was repeated three times.

### 2.6 Lymphocyte proliferation assay

Blood samples were obtained from healthy volunteers. Peripheral blood mononuclear cells (PBMCs) were isolated as described (37). The PBMCs were then counted, centrifuged and resuspended in CIK serum-free culture medium (REGEN-αGEEK) to achieve a specific cell concentration. The PBMCs were then unstained or stained with a final concentration of 2.5 μM CFSE (Biolegend), followed by incubation at 37°C in the dark for 20 min. After terminating the staining by adding complete medium, the PBMCs were centrifuged and resuspended, then incubated for 10 min to inactivate any CFSE that did not enter the cells. After another round of centrifugation, the PBMCs were resuspended in fresh culture medium, counted, and adjusted to the desired cell concentration. The unstained cells and stained PBMCs were seeded in a 12-well culture plate with a final concentration of 5 × 10^5^ cells/ml. The PBMCs were then stimulated with PHA-M (Dakewe) and treated with EVs to inhibit lymphocyte proliferation. After 96 h of incubation at 37°C and 5% CO_2_, the PBMCs were collected, washed with PBS, and analyzed by flow cytometry. The data were processed using FlowJo software to examine cell proliferation in the experimental and control groups. The inhibitory effect of EVs on cell proliferation was evaluated during co-incubation with PBMCs from different individuals and purified at different times.

### 2.7 Study design

This was a pilot, non-randomized, controlled clinical study conducted at Huazhong University of Science and Technology Union Shenzhen Hospital, China, to evaluate the efficacy and safety of nebulization therapy using MSC-EVs for the treatment of chronic cough after COVID-19 infection. A total of 80 patients were enrolled in this study. The first patient was enrolled on February 15, 2023. Due to administrative delays, the study was registered retrospectively at ClinicalTrials.gov (NCT05808400) on April 7, 2023. The research protocol was approved by the Ethics Committee of Huazhong University of Science and Technology Union Shenzhen Hospital, China (KY-2023-033), prior to patient enrollment. Written informed consent was obtained from all participants before treatment. Most patients were enrolled and treated after trial registration. All study procedures were conducted in accordance with the Declaration of Helsinki and relevant institutional requirements.

### 2.8 Inclusion criteria

At the outset of this clinical study, we enrolled patients with chronic cough after COVID-19 infection in accordance with the guidelines provided by the National Health Commission of China. We conducted a comprehensive evaluation of each patient's epidemiological history, clinical symptoms, and nucleic acid test results. Prior to participation, informed consent was obtained from all patients enrolled in the study, and detailed inclusion criteria were established: (1) Trial participants voluntarily participate in this study and sign an informed consent form. (2) At the time of signing the informed consent form, the age of the subject should be ≥18 and ≤ 80 years old, with no gender restrictions. (3) The subject has been diagnosed with COVID-19 (confirmed by positive nucleic acid or antigen test) and has symptoms that have lasted for more than 4 weeks. (4) Negative nucleic acid or antigen test at the time of screening. (5) The subject has had continuous or intermittent coughing, or loss of taste/smell for ≥4 weeks, which did not occur before the onset of COVID-19 infection. (6) No prior treatment with umbilical cord mesenchymal stem cell-derived extracellular vesicles. (7) The patient fully understands the purpose and requirements of this clinical study and is willing to complete all trial procedures according to the study requirements.

### 2.9 Exclusion criteria

(1) Age < 18 or >80 years old. (2) Acute COVID-19 patients. (3) Suspected or confirmed to have severe, active bacterial, fungal, or other infections that may pose a risk when intervention measures are taken, as determined by the researcher. (4) Patients with a history of diagnosed bronchial asthma, cough variant asthma, or chronic cough; patients with other pulmonary diseases such as chronic obstructive pulmonary disease, bronchiectasis, tuberculosis, lung cancer, etc. (5) Any of the following during the screening period: (1) ALT or AST > 3 times the upper limit of normal; (2) eGFR < 60 mL/min. (6) Patients with a history of severe allergies. (7) Patients with uncontrolled severe cardiovascular, cerebrovascular, liver, kidney, endocrine, blood system diseases, and mental illness. (8) Patients with active immunosuppression, immunodeficiency, and use of immunosuppressive drugs. (9) Pregnant and lactating women. (10) Other factors that the researcher deems unsuitable for participation in the study based on clinical considerations.

### 2.10 Nebulization treatment of MSC-EVs

Following ethical approval, patients in the experimental group diagnosed with chronic cough after COVID-19 infection were provided with informed consent and were treated with nebulized MSC-EVs (38). The experimental group received a 5 ml preparation of EVs with a concentration of 1x10^9^ particles/ml (39), while the control group did not receive any EVs treatment during the same period. The nebulized inhalation treatment was administered twice daily, once in the morning and once in the evening, for a continuous period of 5 days, after a skin test was conducted to ensure safety. The frozen EVs preparation was thawed at room temperature and added to the nebulizer, with the treatment lasting for 10 min. Following the nebulization treatment, the patients were assessed by investigators to evaluate the efficacy of the treatment.

### 2.11 Primary outcomes

Cough Evaluation Test (40). This scoring system is designed to evaluate the severity of cough symptoms, with higher scores indicating a worse outcome. The minimum score is 5, while the maximum score is 25. The degree of cough improvement in CET scores was divided into three levels based on the reduction from baseline: Significant effective (reduction≥6), Effective (reduction 2–5), Ineffective (reduction < 2).

### 2.12 Secondary outcomes

Improvement or relief time of symptoms. This indicator is used to evaluate the number of days required to alleviate cough symptoms.

### 2.13 RNA sequencing

Blood samples were collected from chronic cough patients in the MSC-EVs treatment group before treatment (baseline) and at day 14 post-treatment. Peripheral blood mononuclear cells (PBMCs) were isolated as described (37). The raw fastq data were trimmed for adapter sequences using Cutadapt (v0.6.7) and subjected to quality control using FastQC (v0.11.9). The trimmed sequences were then mapped to the GRCh38.p13 genome using STAR (v2.7.9a). The samples were demultiplexed based on barcodes using fastq-multx (v1.4.2), and featureCounts (v2.0.3) was used to count the reads mapped to each gene, resulting in a count matrix. Downstream analysis was performed in R environment version 4.0.5. Quality control steps included: (1). Sample filtering: Outlier samples were removed based on the median absolute deviation (MAD) of the number of detected genes and library size using the scuttle package (v1.0.4). (2). Gene filtering: Genes that were expressed in at least 10% of the samples were retained, while mitochondrial genes, ribosomal genes, and hemoglobin genes were removed. Differential analysis was conducted using DESeq2 (v1.30.1) on the count matrix. The filtering criteria for differentially expressed genes were FDR-corrected *p*-value < 0.05 and absolute log2FoldChange > 1.

Enrichment analysis was performed using the Metascape web tool (https://metascape.org/). Immune cell deconvolution was carried out using the CIBERSORTx web tool (https://cibersortx.stanford.edu/index.php) with the LM22 reference dataset. The results were evaluated using the *Wilcoxon test*.

### 2.14 Statistical analysis

Statistical analyses and graphs were performed and generated using GraphPad Prism software version 10 for Mac (GraphPad Software, San Diego, USA). The normal variables were presented as mean ± SEM (Standard error of the mean) for in vitro experimental data and as mean ± SD for clinical data. Differences between 2 groups were assessed using unpaired Student *t* test. Within-group and between-group changes in CET scores were assessed using the Wilcoxon signed-rank test and Mann–Whitney U test, respectively. Data involving more than 2 groups were assessed using ANOVA. For categorical outcomes, the chi-square test was used. For time to resolution, the unpaired *t*-test was used. *P* < 0.05 was considered statistically significant.

## 3 Results

### 3.1 Characterization of EVs isolated from MSCs

**Figure 1:**
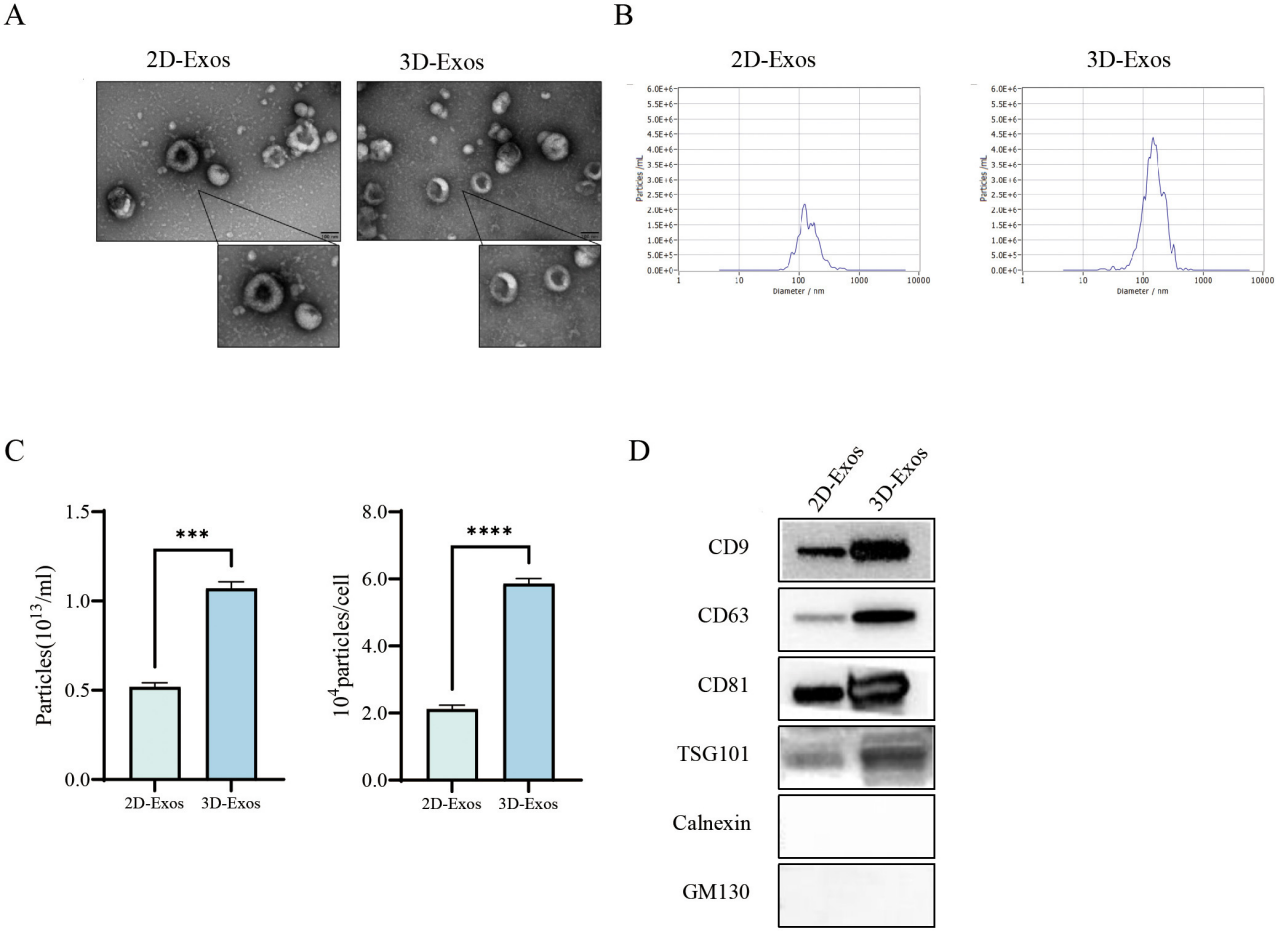
Isolation and characterization of MSC-derived EVs. **(A)** TEM morphology of 2D-EVs and 3D-EVs. **(B)** NTA analysis of 2D-EVs and 3D-EVs. **(C)** Yield of 2D-EVs and 3D-EVs. **(D)** Western blot analysis of 2D-EVs and 3D-EVs protein markers against CD9, CD63, CD81, TSG101, Calnexin and GM130. Continuous variables were described by means ± SEM. ***p < 0.001, ****p < 0.0001.

To compare the differences between 2D-EVs and 3D-EVs, the EVs samples were subjected to TEM and NTA. TEM analysis reveals that compared to 2D-EVs, 3D-EVs exhibit a more pronounced cup-shaped morphology, which is a typical characteristic of EVs (Figure 1A). The NTA results demonstrate that the size distribution of 2D-EVs and 3D-EVs ranges from 50–400 nm and the median diameter of 2D-EVs and 3D-EVs are 129.3 nm and 132.6 nm, respectively, consistent with the characteristics of EVs. However, 3D culture system yields a higher concentration of EVs per unit volume (Figures 1B, C), thus, yields higher levels of particle secretion and EVs secretion from each individual cell (Figure 1C) (41). To confirm the successful isolation of EVs, we conducted western blot using specific antibodies against the well-known EVs markers, such as CD9, CD63, CD81, TSG101 and negative markers such as Calnexin and GM130 (Figure 1D) (42, 43). Taken together, the EVs were isolated successfully. Most importantly, the 3D culture system can significantly increase the production of EVs to meet clinical demands.

### 3.2 3D-EVs promote angiogenesis and cell migration *in vitro*

**Figure 2:**
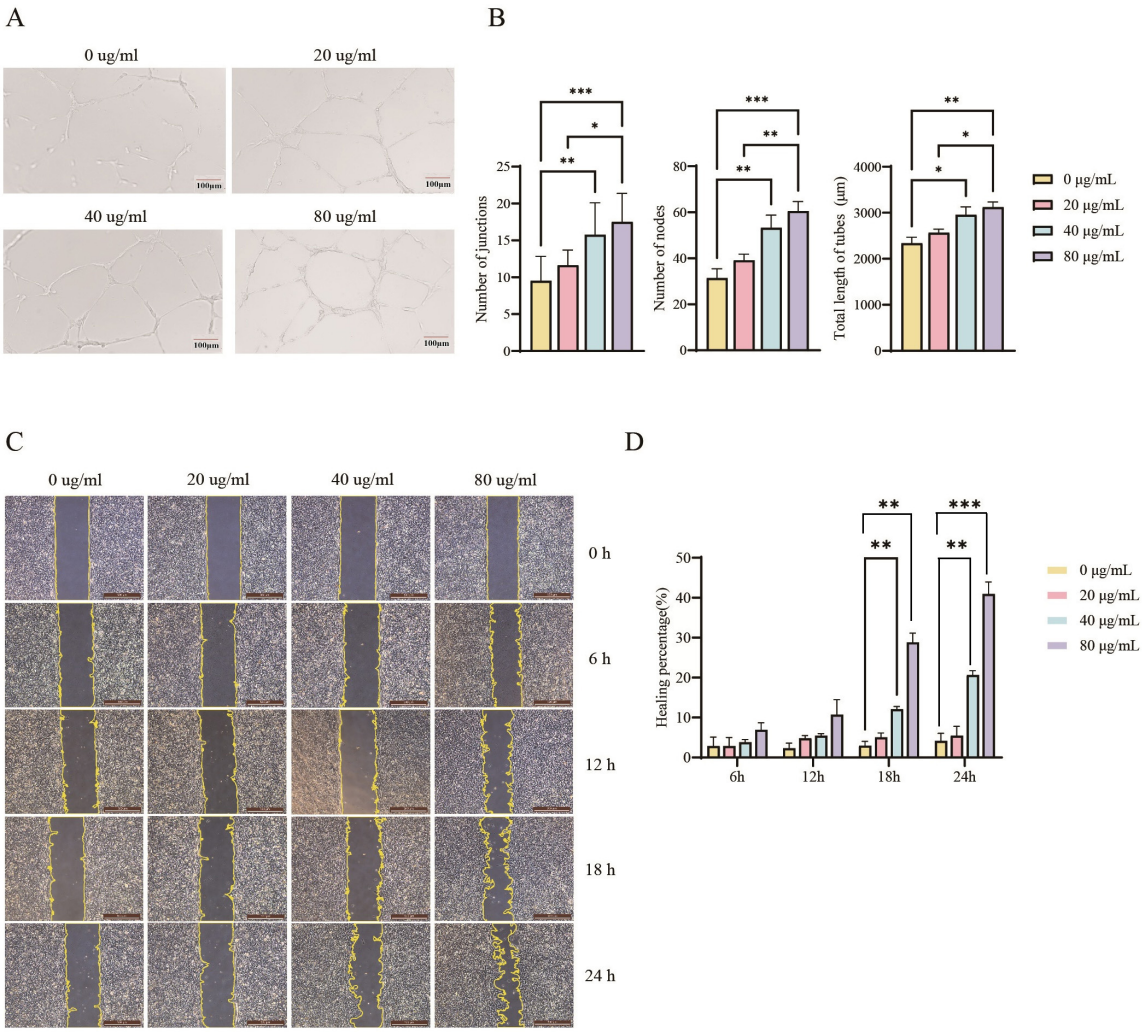
Biological function of 3D-EVs on angiogenesis and cell migration *in vitro*. **(A)** Images of the tube formation of 3D-EVs treated HUVECs for 8 h. **(B)** Quantification analysis of number of junctions, number of nodes and total length of tubes (*n* = 3). **(C)** Images of 3T3 cell migration that treated with 0, 20, 40 and 80 ug/ml 3D-EVs for 0, 6, 12, 18, 24 h. **(D)** Quantification analysis of healing percentage. Continuous variables were described by means ± SEM. *p < 0.05, **p < 0.01, ***p < 0.001.

We next evaluated the biological function of 3D-EVs on angiogenesis *in vitro*, by performing tube formation assay. Primary human umbilical vein endothelial cells (HUVECs) were treated with 3D-EVs at concentrations of 0, 20, 40, and 80 μg/ml 3D-EVs for 8 hours (Figure 2A). As anticipated, we observed an increase in tube nodes, junctions, and tube length of HUVECs in a concentration-dependent manner. The differences were statistically significant at 40 and 80 μg/ml compared to 0 μg/ml 3D-EVs (Figure 2B). Furthermore, we performed a cell scratch assay to assess the migration of mouse embryo fibroblasts in response to 3D-EVs (Figure 2C). The results demonstrated a concentration-dependent manner in cell migration, with a significant increase observed at 40 and 80 μg/ml compared to 0 and 20 μg/ml 3D-EVs after 18 hours (Figure 2D). Taken together, these findings indicate that 3D MSC-EVs exhibit a positive effect on angiogenesis and cell migration *in vitro*.

### 3.3 3D-EVs suppress lymphocyte proliferation in human PBMCs

**Figure 3:**
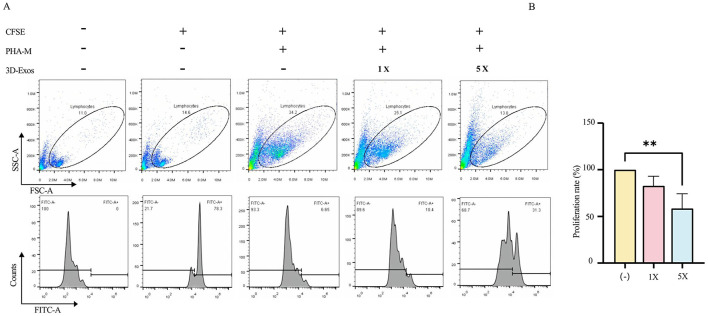
Inhibitory effect of 3D-EVs on lymphocyte proliferation. **(A)** Representative flow cytometry plots of human PBMCs stimulated with PHA-M and treated with 3D-EVs at concentrations of 1x10^9^ particles/ml or 5x10^9^ particles/ml. **(B)** Quantification of lymphocyte proliferation rates after treatment with 3D-EVs. The signals were captured by Flow cytometry and data were processed by FlowJo. **p < 0.01.

We next validated the functionality of 3D-EVs on lymphocyte proliferation using human PBMCs. The PBMCs were stimulated using PHA-M, followed by the addition of two distinct batches of 3D-EVs at concentrations of either 1x10^9^ particles/ml or 5x10^9^ particles/ml. Subsequent monitoring of lymphocyte proliferation was conducted using flow cytometry. Our findings indicate that both batches of 3D-EVs, at either concentration, exert an inhibitory effect on cell proliferation. Moreover, the inhibitory effect was found to be concentration-dependent, with higher concentrations resulting in stronger inhibitory effects. These results provide valuable insights into the potential therapeutic application of MSC-EVs (Figure 3).

### 3.4 Clinical characteristics of the study population

A total of 80 patients were enrolled in this study. Participants were equally divided into the MSC-EVs treatment group and control group. Both groups were treated with the conventional drug compound methoxyphenamine. It was administered orally at a dose of 2 pills per time, three times a day. Clinical characteristics of the study population are shown in (Table 2). MSC-EVs treatment group was given EVs harvested from 3D culture system. The subjects in both the treatment group and the control group were infected with COVID-19 during the same outbreak period from December 2022 to January 2023, suggesting that they may have been exposed to a similar strain of the virus. No significant differences were noted in terms of all clinical characteristics including spirometry parameters and FeNO (Table 2). All of the participants were asked to conduct a Cough Evaluation Test (CET) (Table 3). Both groups had moderate baseline CET scores, which is 14.30 ± 3.24 and 13.80 ± 3.88, respectively, and likewise did not differ significantly between the two groups.

**Table 2:** Clinical characteristics of the study population.

Characteristic	Exosome treatment group	Control group
Number of enrollers	40	40
Gender, *N*%		
Male	17 (42.5%)	17 (42.5%)
Female	23 (57.5%)	23 (57.5%)
Age	41.34 ± 14.37	37.15 ± 10.58
COVID-19 infection time	2022/11/17–2023/1/7	2022/10/21–2023/1/22
Cough duration (weeks)	8.85 ± 1.63	9.15 ± 2.49
Spirometry		
FVC/predicted (%) ≥ 80%	94.51 ± 12.30	95.03 ± 11.80
FEV1/predicted (%)≥ 80%	93.15 ± 10.72	93.47 ± 9.68
FEV1/FVC measured (%)≥ 70%	84.65 ± 8.36	85.18 ± 8.02
MMEF75/25 predicted (%)≥ 65%	77.57 ± 20.0	78.01 ± 19.7
FeNO (pbb)	17.55 ± 15.82	16.87 ± 12.10
CET	14.30 ± 3.24	13.80 ± 3.88

Both groups received conventional drug treatment for cough suppression, expectoration, and anti-allergy (Compound Methoxyphenamine, orally taken 2 capsules once, three times a day). FVC, Forced Vital Capacity; FEV1, Forced Expiratory Volume in One Second; FEV1/FVC, The ratio of FEV1 to FVC; MMEF75/25, Mean Mid-Expiratory Flow 75/25; FeNO, Fractional Exhaled Nitric Oxide.

**Table 3:** Cough evaluation test (CET).

Please read each question carefully to assess your condition at present and answer by “√” the response that best applies to you	None	Seldom	Sometimes	Often	All of the time
	1	2	3	4	5
How frequently did you cough during the day?	1	2	3	4	5
Have your cough disturbed your sleep?	1	2	3	4	5
Did you have intense cough?	1	2	3	4	5
Have your cough interfered with your daily life?	1	2	3	4	5
Have your cough made you feel anxious or depressive?	1	2	3	4	5

### 3.5 3D-EVs treatment demonstrates rapid onset of efficacy in alleviating chronic cough symptoms

**Figure 4:**
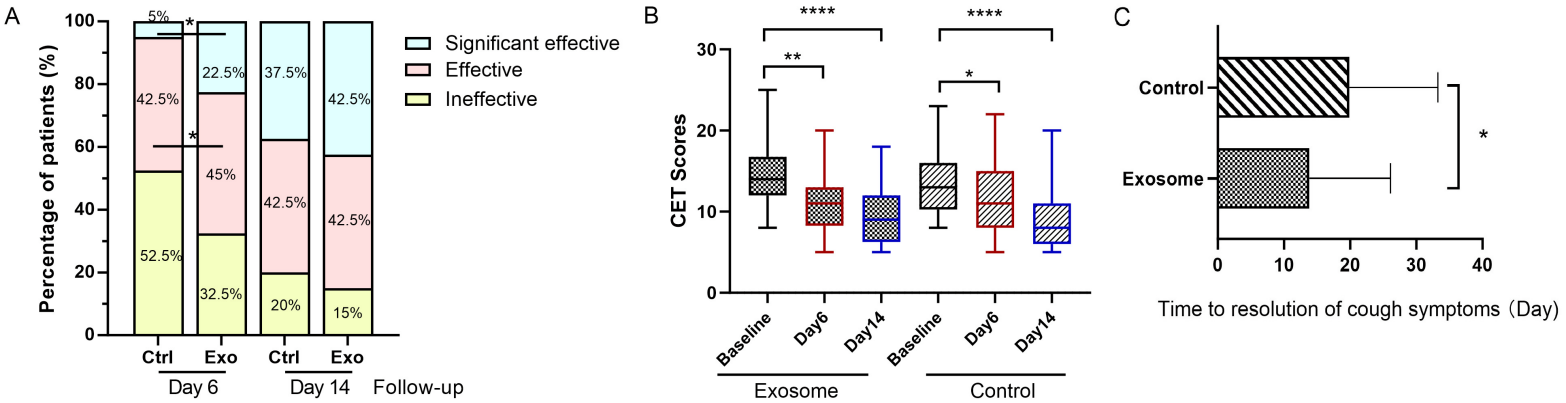
Clinical efficacy of nebulized 3D-EVs for post-COVID-19 chronic cough. **(A)** Efficacy categorization by Cough Evaluation Test (CET) score improvement at Day 6 and Day 14. **(B)** Median CET score changes from baseline to Day 6 and Day 14. **(C)** Time to cough symptom resolution. Statistical analyses: chi-square test for **(A)**, Wilcoxon signed-rank test for **(B)**, unpaired *t*-test for **(C)**. *P < 0.05, **P < 0.01, ****P < 0.0001.

At the 6-day follow-up, the MSC-EVs treatment group showed a significantly higher rate of significant improvement (22.5% vs. 5.0%, *P* = 0.023) and a significantly higher total effective rate (effective + significant effective) than the control group (67.5% vs. 47.5%, *P* = 0.035). By day 14, both groups achieved similar total effective rates (85% vs. 80%, *P* = 0.452) (Figure 4A). By day 14, the MSC-EVs group had 42.5% significant improvement and 42.5% improvement (total effective rate 85%), while the control group had 37.5% and 42.5%, respectively (total effective rate 80%), with no significant difference between the two groups (*P* > 0.05) (Figure 4A).

The Cough Evaluation Test score decreased from baseline to day 6 and day 14 in both groups (Figure 4B). At day 6, the reduction from baseline was significantly greater in the treatment group than in the control group (*P* < 0.05). At day 14, both groups showed comparable improvements (Figure 4B). The time to resolution of cough symptoms was significantly shorter in the MSC-EVs group, with a mean resolution time of 13.83 ± 12.26 days compared to 19.90 ± 13.37 days in the control group (*P* = 0.037) (Figure 4C). These results indicate that MSC-EVs treatment accelerates early symptom relief and shortens the overall duration of cough, while later follow-up at day 14 efficacy in terms of total effective rate is comparable to conventional therapy.

### 3.6 Exploratory immunomodulatory analyses of MSC-EVs in chronic cough treatment

**Figure 5:**
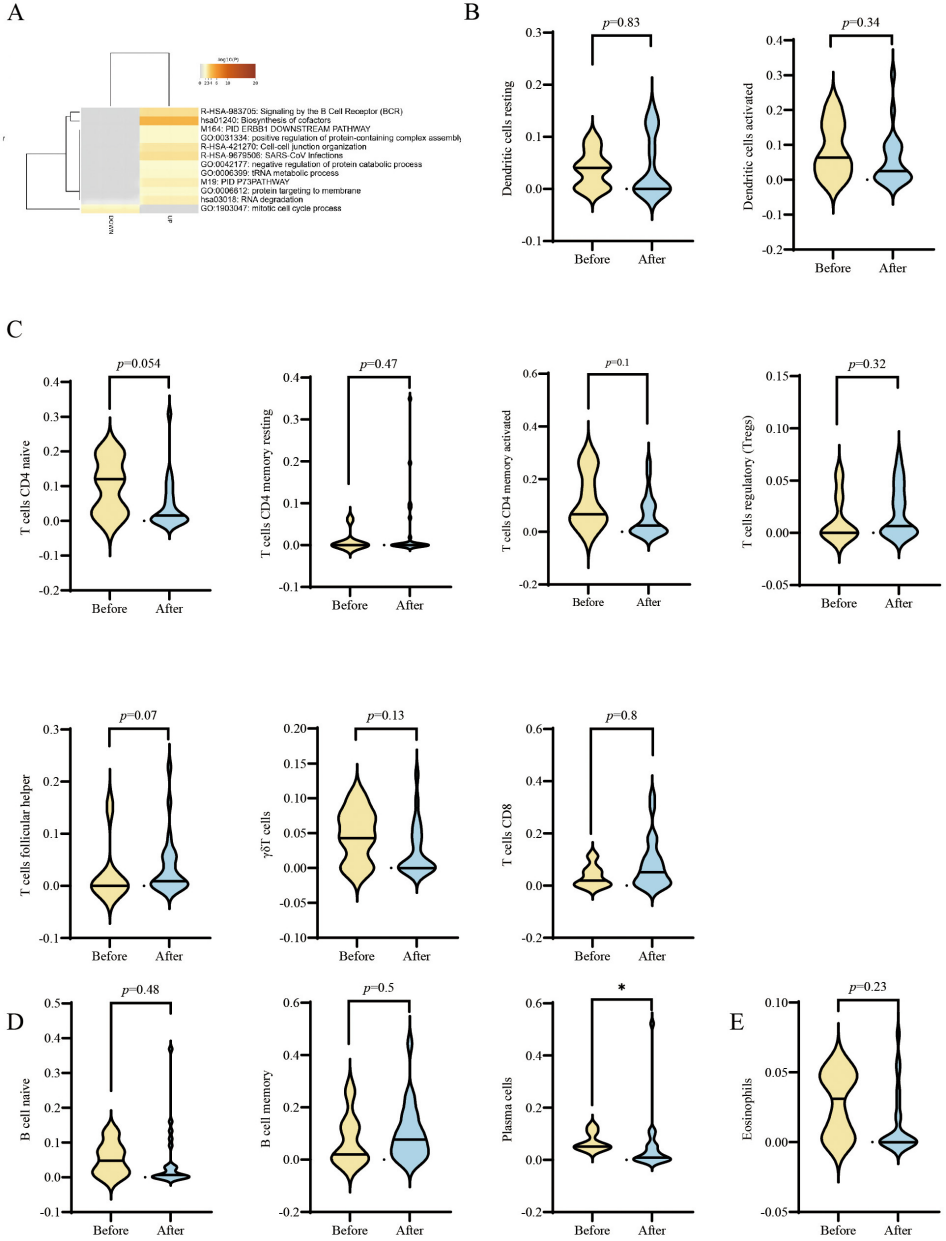
Exploratory immunomodulatory analyses of MSC-EVs. **(A)** Heat map of significantly altered pathways before and after 3D-EVs treatment in PBMCs (FDR, *P* < 0.05). **(B–E)** Immunophenotyping of PBMCs. Results are shown as violin plots for the distribution and change in each of the parameter studied. The median is represented by a bold dashed line. *P* values, by Wilcoxon test, display the levels of significance between before and after treatment. *P < 0.05.

RNA sequencing and immunophenotyping data from PBMCs of MSC-EVs-treated patients were analyzed to investigate potential immunomodulatory effects (Figure 5). RNA sequencing data revealed upregulation of B cell receptor (BCR) pathway, cofactor biosynthesis and ErbB1(EGFR) downstream pathways in PBMCs post-treatment compared to baseline (Figure 5A, FDR < 0.05), suggesting potential alterations in B cell-related signaling and immune-regulatory pathways (44). Downregulation of the mitotic cell cycle process pathway is consistent with inhibition of cell proliferation observed *in vitro* (Figure 3).

Immunophenotyping showed directional trends in several immune cell subsets. The median percentages of resting dendritic cells (DCs), activated DCs, CD4+ T cells, and B cells decreased after 3D-EVs treatment, while regulatory T cells (Tregs) showed an increasing trend and eosinophils showed a decreasing trend (Figures 5B–E). Notably, plasma cells showed a statistically significant reduction (*P* < 0.05, Figure 5D). However, due to limited sample size and inter-individual variability, most of these changes did not reach statistical significance.

### 3.7 MSC-EVs treatment demonstrates safety and tolerability in chronic cough patients

All participants tolerated the MSC-EVs treatment nebulization well, no abnormal signs, such as, temperature, heart rate or respiratory rate were observed during the treatment, and no adverse events were reported. The hematological and biochemical parameters (white blood cell count, lymphocyte count, monocyte count, C-reactive protein, alanine aminotransferase, aspartate aminotransferase, and creatinine) before and after treatment are summarized in Table 4. These data indicate there is no significant impact on hematological and biochemical parameters, which imply MSC-EVs treatment for chronic cough appears to be safe.

**Table 4:** Safety outcomes before and after nebulization treatment.

Before (Day 0)							After (Day 1)						
WBC (x10^9^/L)	LYMPH# (x10^9^/L)	MONO# (x10^9^/L)	CRP (mg/L)	ALT (U/L)	AST (U/L)	CRE (μmol/L)	WBC (x10^9^/L)	LYMPH# (x10^9^/L)	MONO# (x10^9^/L)	CRP (mg/L)	ALT (U/L)	AST (U/L)	CRE (μmol/L)
6.65 ± 1.48	2.08 ± 0.47	0.40 ± 0.12	1.42 ± 2.81	22.10 ± 19.91	19.05 ± 6.04	66.32 ± 14.65	6.61 ± 2.07	2.13 ± 0.47	0.39 ± 0.12	0.58 ± 2.96	22.20 ± 19.12	19.75 ± 6.22	67.26 ± 15.33

WBC, White Blood Cells; LYMPH#, Lymphocyte count; MONO#, Monocyte count; CRP, C-Reactive Protein; ALT, Alanine Aminotransferase; AST, Aspartate Aminotransferase; CRE, Creatinine.

## 4 Discussion

The therapeutic potential of MSC-EVs is increasingly recognized in various medical fields. MSC-EVs possess unique properties, including the ability to transfer bioactive cargos such as proteins, mRNAs, and microRNAs, which makes them attractive candidates for therapeutic interventions (45–47). Several other clinical trials have demonstrated the capacity of MSC-EVs to reduce inflammatory factor levels and modulate immune responses in various types of COVID-19 (NCT04384445, USA; NCT04491240, Russia). A single-center prospective, open-label, single arm interventional study on aerosolized ChipEXO™ for severe COVID-19 pneumonia showed promising results (39). Thirteen patients received the treatment without any adverse reactions. The survival rate was 84.6%, and most patients experienced improvements in respiratory parameters and inflammatory markers. Our clinical trial findings suggest a favorable therapeutic efficacy and safety profile for MSC-EVs treatment (Figure 4). Treatment with MSC-EVs demonstrated a significantly faster improvement in chronic cough symptoms at the 6-day follow-up compared to the control group, although the long-term (day 14) efficacy was comparable between the two groups. Notably, this rapid onset of action (Day 6) may be clinically meaningful for patients suffering from persistent post-COVID cough, consistent with previous reports that MSC-EVs can rapidly modulate immune responses (31, 32). The comparable long-term outcomes may be attributable to several factors, including the gradual effects of conventional antitussive medication and spontaneous improvement.

Previous studies have suggested that MSCs can promote Treg expansion and reduce eosinophilic inflammation through various mechanisms (12, 48). Our RNA sequencing and immunophenotyping data from patients' PBMCs show that MSC-EVs treatment was associated with directional trends including decreased proportions of B cells and CD4+ T cells, inhibition of cell proliferation, expansion of Tregs, and reduction of eosinophils (Figure 5). The absence of statistical significance for the majority of these changes is probably explained by the modest sample size and considerable variability among individuals. Notably, a statistically significant reduction in plasma cells was observed (Figure 5D). Together with the rapid clinical improvement shown in Figure 4, these exploratory findings suggest that the immunomodulatory effects of 3D-EVs may collectively contribute to the early alleviation of chronic cough symptoms (48). Larger cohort studies are needed to confirm these observations.

Moreover, our results reveal that 3D-EVs not only exhibit typical EVs characteristics but also have a higher concentration per unit volume compared to 2D-EVs (Figure 1), suggesting a superior production efficiency which could meet clinical demands. The increased production of EVs in the 3D culture system is critical as it can facilitate the large-scale production of EVs for therapeutic applications (49). In addition to yield, the 3D culture microenvironment may influence MSC behavior and EV properties by better preserving cell-cell and cell-matrix interactions. Previous studies have suggested that 3D culture or bioreactor-based systems can alter EV secretion and cargo profiles and may enhance the biological functions of MSC-EVs, including reduced senescence and enhanced capabilities in migration, angiogenesis, and anti-inflammatory responses (50, 51). Thus, the 3D culture system may have potential advantages for both scalable EV production and functional EV generation. As far as we know, we are the first to utilize 3D-cultured MSC-EVs to treat post-COVID chronic cough. Nevertheless, because this study did not directly compare the molecular cargo of 2D-EVs and 3D-EVs, further proteomic, transcriptomic, and functional analyses are needed to clarify the qualitative advantages of 3D-EVs.

In addition, the *in vitro* experiments demonstrate the potential of 3D-EVs in promoting angiogenesis and cell migration (Figure 2), crucial processes in tissue repair and regeneration. This observation aligns with the known role of MSCs in tissue repair, further supporting the therapeutic potential of MSC-EVs (52). Importantly, our study confirmed that 3D-EVs also carry the ability to suppress lymphocyte proliferation in human PBMCs (Figure 3), as MSCs do, suggesting their potential role in modulating immune responses (53). This anti-proliferative effect is consistent with the known immunomodulatory functions of MSCs and their EVs (54).

Several limitations of this study should be acknowledged. For instance, the absence of a placebo nebulization for the control group may introduce bias, so the observed efficacy difference at day 6 should be interpreted with caution. Additionally, our findings are specific to post-COVID chronic cough and cannot be extrapolated to other etiologies. Moreover, the relatively small sample size limited statistical power for immunophenotypic analyses; most immune cell subsets showed only directional trends. In addition, we did not perform a detailed cargo comparison between 2D and 3D-EVs, which limits our understanding of the qualitative advantages of the 3D culture system.

Overall, our study provides initial evidence supporting the therapeutic potential of MSC-EVs in treating post-COVID chronic cough. Future studies should use larger cohorts and placebo-controlled designs to validate the clinical efficacy of 3D-EVs, clarify their precise molecular mechanisms and further explore their potential in treating other respiratory diseases.

## 5 Conclusion

In summary, this pilot study demonstrates that nebulized 3D-EVs provide faster early relief of post-COVID chronic cough (superior at day 6, comparable at day 14) with good tolerability. Exploratory analyses suggested potential modulation of B cell-related immune responses, including altered B cell receptor signaling and reduced plasma cells. These findings support the feasibility of MSC-EVs as a novel therapeutic candidate for post-COVID chronic cough.

## Data Availability

The datasets presented in this study can be found in online repositories. The names of the repository/repositories and accession number(s) can be found below: https://www.ncbi.nlm.nih.gov/, GSE334259.
